# Supplementary material for: Sex-specific fear acquisition following early life stress is linked to amygdala and hippocampal purine and glutamate metabolism
Source: Commun Biol. 2024 Dec 20;7:1684. doi: 10.1038/s42003-024-07396-8 (PMC11659400; doi:10.1038/s42003-024-07396-8)
Supplement: Supplementary file 2 — Supplemental Material [file 42003_2024_7396_MOESM2_ESM.pdf]

Sex-specific fear acquisition following early life stress is linked to amygdala glutamate metabolism

Joeri Bordes, Thomas Bajaj, Lucas Miranda, Lotte van Doeselaar, Lea Maria Brix, Sowmya Narayan, Huanqing Yang, Shiladitya Mitra, Veronika Kovarova, Margherita Springer, Karin Kleigrewe, Bertram Müller-Myhsok, Nils C. Gassen, Mathias V. Schmidt

Supplemental materials

Supplemental Materials and Methods

Early life stress paradigm: limited bedding and nesting

Early life stress (ELS) was performed using the limited bedding and nesting (LBN) paradigm to induce chronic stress towards the mother and pups during P02 to P09, as previously described by Rice et al. [1]. At P02, all litters were transferred to new IVCs and randomly assigned to the non-stressed (NS) or stressed (LBN) condition. If necessary, the litters were culled to a maximum of 10 animals per litter. The LBN litters were placed on a stainless-steel mesh (McNichols) and were provided with limited nesting material (1/2 square of Nestlets, Indulab). The NS animals were placed in an IVC with a standard amount of bedding material and were provided with a sufficient amount of nesting material (2 squares of Nestlets). All litters were left undisturbed until P09, after which they returned to standard housing conditions. The pups were weaned in same-sex groups with a maximum of four animals per cage.

Physiological measurements

One week following the behavioral tests, adult animals were weighed and subsequently sacrificed by decapitation, after which trunk blood was collected in EDTA-coated microcentrifuge tubes (Kabe Labortechnik, Germany) and directly transferred to ice. Samples were centrifuged at 4°C for 15min at 8.000 rpm, after which plasma was removed and kept transferred for storage at -80°C. A separate cohort of mice was used to obtain corticosterone (CORT) measurements directly after the stress at P09. On the morning of P09, litters were kept in their cage, while first the mother was sacrificed, and subsequently

the pups were sacrificed, keeping them in their nest as long as possible to minimize the influence of acute stress exposure. Trunk blood was collected and processed as described for adult blood samples. Plasma CORT levels were measured in duplicates using radioimmunoassay following the manufacturer's protocol (MP Biomedicals, Eschwege, Germany). Adrenals were dissected and kept at 4°C in saline (0.9% NaCl) until further processing, which included the removal of all surrounding fat tissue and weighing. The relative adrenal weight was calculated by dividing the total body weight before sacrifice by the total adrenal weight, including the adrenals from both sides.

In-situ hybridization of *FKBP5*

In brief, the animals were either sacrificed directly after the stress exposure at P09 or in adulthood at 2 months of age. After decapitation, the brains were removed and snap-frozen using 2-methyl butane (kept on dry ice) and stored at -80°C until further use. Brains were sliced using a cryostat in 20 µm sagittal sections, which resulted in a series of the BLA and dHIP slides that were thaw-mounted on Super Frost Plus Slides and stored at -20°C. The *in-situ* hybridization sections were removed from -20°C, left to dry at room temperature, fixated with 4% paraformaldehyde, and subsequently dehydrated using a series of increasing concentrations of ethanol. Then, the hybridization buffer was equally spread out over the different slides containing the radioactive ^{35}S-UTP-labeled *FKBP5* riboprobe and incubated overnight at 55°C. On the next day, the sections were rinsed, incubated with RNAse A, desalted, and dehydrated, after which the radioactive slides were exposed to Kodak Biomax MR films (Eastman Kodak Co., Rochester, NY) and developed after an exposure time of 12 days. Films were digitized, and the regions of interest were identified using the mouse brain atlas (https://developingmouse.brain-map.org/static/atlas). The expression was determined by optical densitometry with the ImageJ software (NIH, Bethesda, MD, USA). The expression was averaged per brain region per animal and subtracted by the background signal of a nearby structure that did not express the *FKBP5* gene. A distinction was made between important

subregions of the dorsal HIP, in which separate measurements were obtained for the CA1, CA2-3, and the dentate gyrus (DG).

Fear conditioning

Fear acquisition

The fear acquisition consisted of placing the mice into a cube-shaped fear conditioning chamber (Bioseb, France) with a metal grid floor to provide electric shocks. At the start of the test, the chamber light was switched on, and after an initial habituation time of 3 min, the mice were exposed to five conditioned-unconditioned stimulus pairings (auditory conditioning stimulus: 30 sec, 9kHz, 80dB tone & unconditioned stimulus 0.5 sec, 0.6mA foot shock) with an inter-trial interval (ITI) of 5 mins. 1 min after the last foot shock the animals were returned to their home cage. Before and after each trial, the conditioning chamber was thoroughly cleaned with 70% EtOH. The calculation of the mean freezing statistics was performed using the average of tones 2-5, leaving out the first tone, as no shock history was present at that moment. The mean ITI freezing was calculated using all four ITIs.

Contextual fear memory

Contextual fear memory was performed 24 hours after initial fear acquisition. The same setup was used as by fear acquisition, except that no conditioned-unconditioned stimulus protocol was executed. The test endured for a total of 5min in which only the chamber light was switched on, and again before and after each trial, the conditioning chamber was thoroughly cleaned with 70% EtOH.

Auditory fear memory

The consolidation of auditory fear memory was performed two days after the fear acquisition. The set-up was replaced by a novel and neutral context, which differed in material (plexiglass), shape (circular), and surface texture, as no grid was present at the bottom of the set-up. In addition, the cleaning solution was

changed in odor, using 1% acetic acid. This allowed for measuring the fear response specifically towards the tones, without the interference of the context. The chamber light was switched on at the start of the test, after which mice were left undisturbed for an initial 1 min habituation phase. Then, the mice were exposed to the same tones as heard in the fear acquisition (30 sec, 9kHz, 80dB) 15 times with a 1.5 min ITI. 1 min after the last tone the animals were returned to their home cage. The mean tone and ITI freezing were calculated using all tones and ITIs.

Metabolomics

Extraction of polar metabolites from mouse BLA, dHIP and vHIP tissue samples

To extract polar metabolites from mouse (BLA, dHIP, and vHIP tissue samples; female: NS n=10, LBN n=10 & male: NS n=11, LBN n=11), a sample extraction buffer consisting of methyl tert-butyl ether (MTBE, Sigma-Aldrich, 650560), methanol (Carl Roth, P717.1), and water (Biosolve, 232141), all of LC-MS grade, in a volumetric ratio of 50:30:20 [v:v:v] was used. The sample extraction buffer contained the following internal standards: U-13C15N-labeled amino acids at a final concentration of 0.25 µM (prepared as 2.5 mM in 0.1 N HCl, Cambridge Isotope Laboratories, MSK-A2-1.2), citric acid d4 at 0.02 µg/mL (dissolved at 100 µg/mL in H2O, Sigma-Aldrich, 485438-1G), ATP 13C10 at 0.1 µg/mL (dissolved at 1 mg/mL in 5 mM Tris-HCl, Sigma-Aldrich, 710695), AMP 13C10, 15N5 at 0.1 µg/mL (dissolved at 1 mg/mL in 5 mM Tris-HCl, Sigma-Aldrich, 650676), ADP 15N5 at 0.1 µg/mL (dissolved at 1 mg/mL in 5 mM Tris-HCl, Sigma-Aldrich, 741167), and EquiSPLASH™ LIPIDOMIX at 0.02 µg/mL (prepared as 100 µg/mL, Avanti Polar Lipids, 30731).

The sample extraction buffer was freshly prepared and cooled to -20°C. Subsequently, 1 mL of the chilled sample extraction buffer was added to the homogenized tissue, which had been preprocessed with 5 mm metal balls in a TissueLyser (Qiagen, TissueLyser LT) for 1 minute at 25 Hz. The mixture was then incubated at 4°C for 30 minutes with agitation at 1500 rpm using a thermomixer (VWR, Thermal Shake lite).

Following incubation, metal balls were removed, and the samples underwent centrifugation (Thermo Scientific, Fresco 21) at 4°C for 10 minutes at 21,000 x g, resulting in the transfer of the cleared supernatant to a 2 mL tube. To this supernatant, 200 µL of MTBE and 150 µL of water were added, followed by incubation at 15°C for 10 minutes with agitation at 1500 rpm in a thermomixer. A subsequent centrifugation at 15°C for 10 minutes at 16,000 x g facilitated phase separation. Approximately 650 µL of the upper lipid-containing phase was transferred to a separate 1.5 mL tube (not included in this study). The remaining polar metabolite extract, approximately 600 µL in volume after removal of the residual lipid phase, was dried using a SpeedVac concentrator (Eppendorf, 5301 Vacufuge). The resulting dried metabolite pellets were subsequently stored at -80°C until further analysis.

HILIC-MS for profiling of metabolites in mouse brain tissue

The samples were dissolved using 100 µL of 70% MeOH (v/v). After 10 min of shaking at 1100 rpm at 10°C with a ThermoMixer® C (Eppendorf AG, Hamburg, Germany), the samples were centrifuged at 13 000 rpm for 10 min at 10°C with Centrifuge 5424 R (Eppendorf AG, Hamburg, Germany). The clear supernatant was transferred to a 1.5 mL glass vial with insert. A QC-sample was pooled by combining 5 µL of each sample.

The untargeted analysis was performed using a Nexera UHPLC system (Shimadzu, Duisburg, Germany) coupled to a Q-TOF mass spectrometer (TripleTOF 6600 AB Sciex, Darmstadt, Germany). Separation of the samples was performed using a UPLC Premier Amide 2.1 × 100 mm, 1.7 µm analytic column (Waters, Eschborn, Germany) with a 400 µL/min flow rate. The mobile phase was 5 mM ammonium acetate in water (eluent A) and 5 mM ammonium acetate in acetonitrile/water (95/5, v/v) (eluent B). The gradient profile was 100% B from 0 to 1.5 min, 60% B at 8 min and 20% B at 10 min to 11.5 min and 100% B at 12 to 15 min. A volume of 5 µL per sample was injected. The autosampler was cooled to 10 °C and the column oven heated to 40 °C. The samples were measured in a randomized order and in the Information

Dependent Acquisition (IDA) mode. MS settings in the positive mode were as follows: Gas 1 55, Gas 2 65, Curtain gas 35, Temperature 500 °C, Ion Spray Voltage 5500, declustering potential 80. The mass range of the TOF MS and MS/MS scans were 50–2000 m/z and the collision energy was ramped from 15–55 V. MS settings in the negative mode were as follows: Gas 1 55, Gas 2 65, Cur 35, Temperature 500 °C, Ion Spray Voltage –4500, declustering potential –80. The mass range of the TOF MS and MS/MS scans were 50–2000 m/z and the collision energy was ramped from –15–55 V.

The "msconvert" from ProteoWizard [2] was used to convert raw files to mzXML (de-noised by centroid peaks). The bioconductor/R package xcms [3] was used for data processing and feature identification. More specifically, the matchedFilter algorithm was used to identify peaks (full width at half maximum set to 7.5 seconds). Then the peaks were grouped into features using the "peak density" method [3]. The area under the peak was integrated to represent the abundance of features. The retention time was adjusted based on the peak groups present in most samples. To annotate features with names of metabolites, the exact mass and MS2 fragmentation pattern of the measured features were compared to the records in HMBD [4] and the public MS/MS spectra in MSDIAL [5], referred to as MS1 and MS2 annotation and their standards, respectively. Missing values were imputed with half of the limit of detection (LOD) methods, i.e., for every feature, the missing values were replaced with half of the minimal measured value of that feature in all measurements. To confirm a MS2 spectra is well annotated, we manually reviewed our MS2 fragmentation pattern and compared it with records in the public database or previously measured reference standards as well as to SIRIUS [6] to evaluate the correctness of the annotation.

Annotated features were subjected to a metabolite set enrichment analyses, MSEA, using KEGG pathway-based library [7] with the only use of metabolite sets containing at least 2 entries in MetaboAnalyst 5.0 [8–11]. The metabolic analysis investigates the expression of a set of metabolites using two different settings, including a HILIC negative mode and a HILIC positive mode. These different HILIC settings can reveal partially different metabolites, and are merged to obtain complete list of metabolites.

References

1. Rice, C. J., Sandman, C. A., Lenjavi, M. R. & Baram, T. Z. A novel mouse model for acute and long-lasting consequences of early life stress. Endocrinology 149, 4892–4900 (2008).
2. Kessner, D., Chambers, M., Burke, R., Agus, D. & Mallick, P. ProteoWizard: open source software for rapid proteomics tools development. Bioinformatics 24, 2534–2536 (2008).
3. Smith, C. A., Want, E. J., O'Maille, G., Abagyan, R. & Siuzdak, G. XCMS: Processing Mass Spectrometry Data for Metabolite Profiling Using Nonlinear Peak Alignment, Matching, and Identification. Anal Chem 78, 779–787 (2006).
4. Wishart, D. S. et al. HMDB 4.0: the human metabolome database for 2018. Nucleic Acids Res 46, D608–D617 (2018).
5. Tsugawa, H. et al. MS-DIAL: data-independent MS/MS deconvolution for comprehensive metabolome analysis. Nat Methods 12, 523–526 (2015).
6. Dührkop, K. et al. SIRIUS 4: a rapid tool for turning tandem mass spectra into metabolite structure information. Nat Methods 16, 299–302 (2019).
7. Kanehisa, M. et al. KEGG for linking genomes to life and the environment. Nucleic Acids Res 36, D480–D484 (2007).
8. Xia, J. & Wishart, D. S. MSEA: a web-based tool to identify biologically meaningful patterns in quantitative metabolomic data. Nucleic Acids Res 38, W71–W77 (2010).
9. Chong, J. et al. MetaboAnalyst 4.0: towards more transparent and integrative metabolomics analysis. Nucleic Acids Res 46, W486–W494 (2018).
10. Pang, Z. et al. Using MetaboAnalyst 5.0 for LC–HRMS spectra processing, multi-omics integration and covariate adjustment of global metabolomics data. Nat Protoc 17, 1735–1761 (2022).
11. Pang, Z. et al. MetaboAnalyst 5.0: narrowing the gap between raw spectra and functional insights. Nucleic Acids Res 49, W388–W396 (2021).

Supplemental Figures

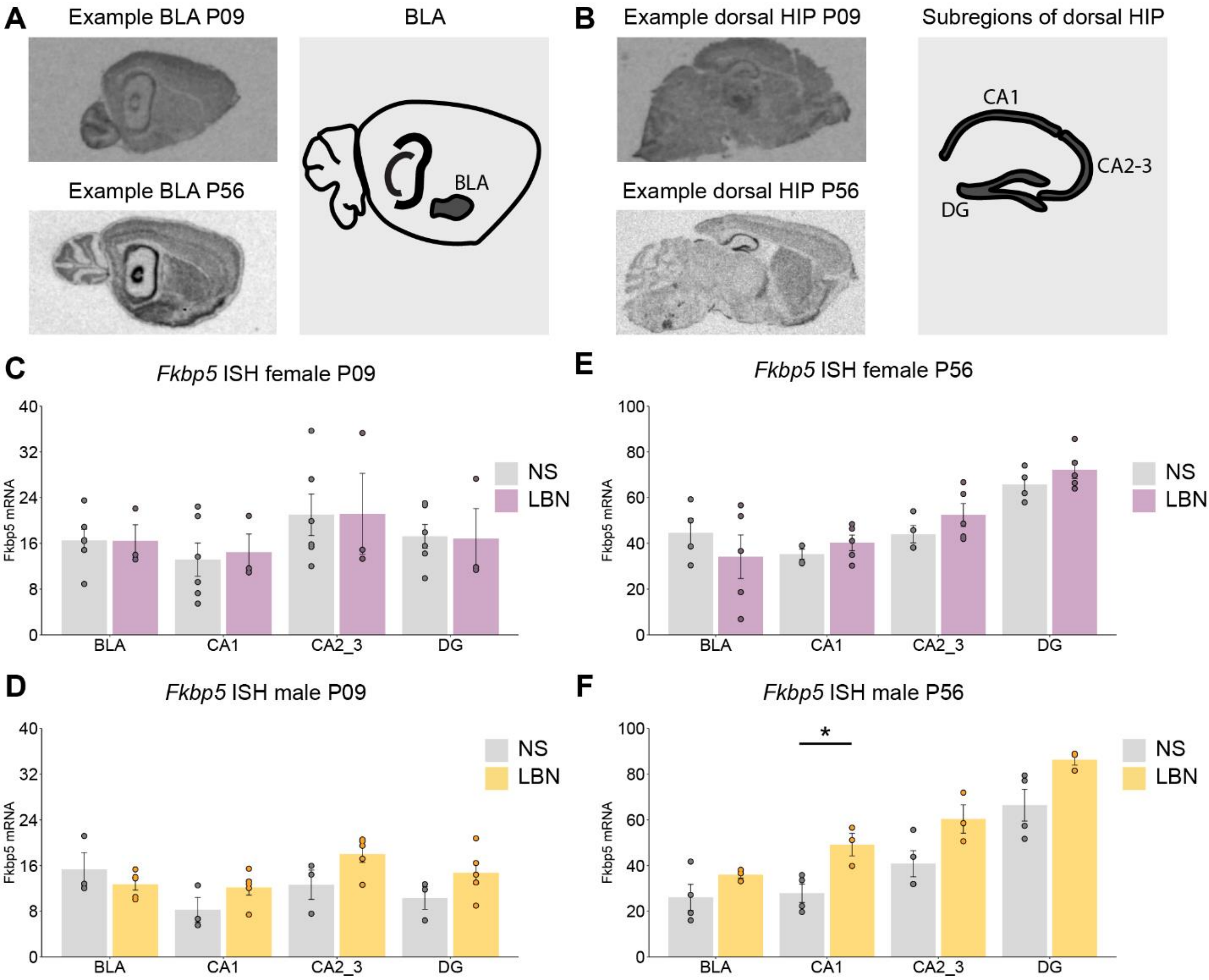

Supplemental Figure 1. In-situ hybridization of *FKBP5* mRNA in the BLA and dorsal hippocampal subregions of non-stressed (NS) and stressed (LBN) mice. A) In-situ hybridization (ISH) scan of *FKBP5* mRNA expression at P09 and P56 in the BLA. **B)** In-situ hybridization scan of *FKBP5* mRNA expression at P09 and P56 in the subregions of the dorsal HIP. **C)** No differences were observed in females at P09 in the BLA (T(7)=0.03, p=0.98), CA1 (T(7)=-0.28, p=0.79), CA2-3 (Wx=11, p=0.71), and DG (T(7)=0.09, p=0.93). **D)** No differences were observed in males at P09 in the BLA (T(6)=1.03, p=0.34), CA1 (T(6)=-1.66, p=0.15), CA2-3 (T(6)=-1.98, p=0.096), and DG (T(6)=-1.49, p=0.19). **E)** No differences were observed in females at P56 in the BLA (T(7)=0.86, p=0.42), CA1 (T(7)=-1.14, p=0.29), CA2-3(T(7)=-1.27, p=0.25), and DG (T(7)=-1.21, p=0.27). **F)** A significant difference for elevated *FKBP5* mRNA expression was observed in the LBN condition for the males at P56 in the CA1 (T(5)=-3.38, p=0.020), but not in the BLA (T(5)=-1.44, p=0.21), CA2-3 (T(5)=-2.30, p=0.070), and DG (We(3.67)=-2.69, p=0.056). The bar graphs are presented as mean ± standard error of the mean and all individual samples as points. Panel C represent female P09 NS (n=6, female LBN

(n=4). Panel D represent male P09 NS (n=3, male LBN (n=5). Panel E represent female P56 NS (n=4), female LBN (n=5). Panel F represent male P09 NS (n=4), male LBN (n=3).

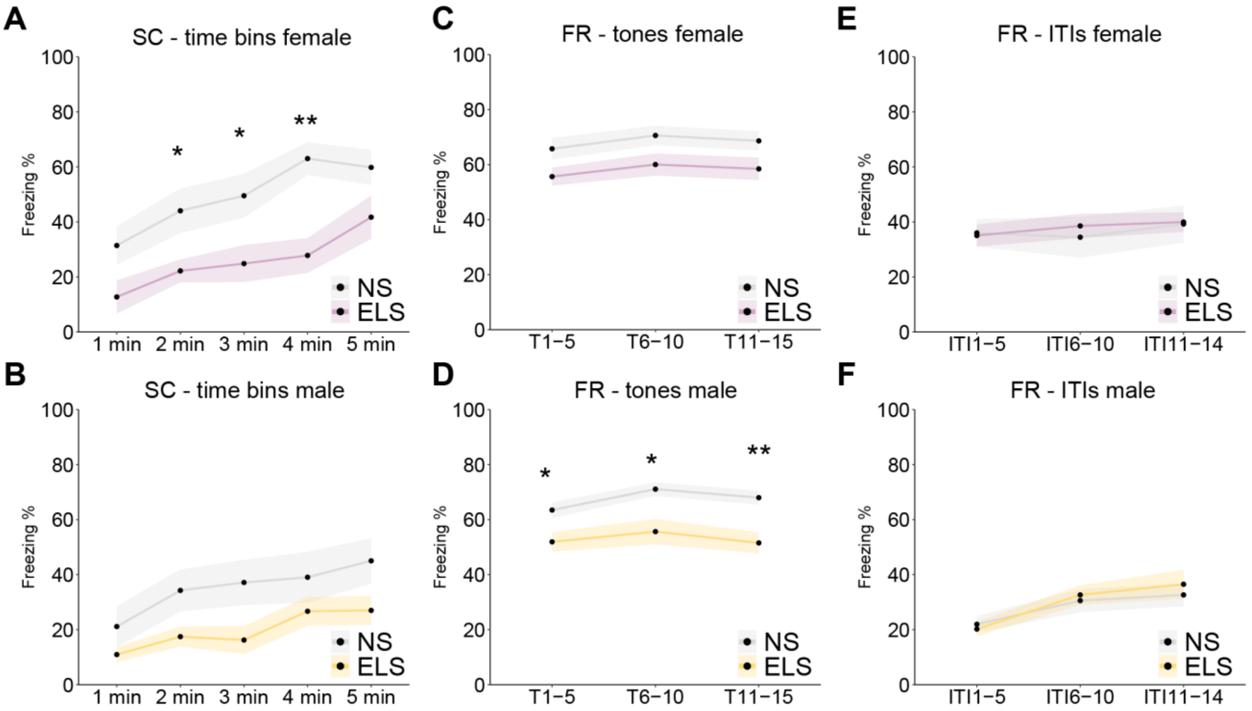

Supplemental Figure 2. Time bin data for contextual and retrieval fear memory. A) The freezing behavior during 1min bins of the contextual fear memory in females. A significant main effect was observed using the two-way ANOVA on stress (F(1,85)=31.61, p<0.0001), and tones (F(4,85)=5.54, p=0.0005), but not on stress*tones (p=0.70). Post-hoc analysis using BH revealed a significant reduction of freezing in LBN females compared to NS for 2min (p=0.047), 3min (p=0.047), and 4min (p=0.004), but not 1min (p=0.069), and 5min (p=0.094). **B)** No significant main effect was observed using the Kruskal Wallis test (p>0.15). **C)** The freezing behavior during the auditory fear retrieval tones binned per 5 tones in females. A significant main effect was observed using the two-way ANOVA on stress (F(1,42)=11.59, p=0.001), but not for tones, or stress*tones (p>0.46). Post-hoc analysis using BH revealed no further significance between stress conditions (p=0.077). **D)** The freezing behavior during the auditory fear retrieval tones binned per 5 tones in males. A significant main effect was observed using the two-way ANOVA on stress (F(1,54)=27.96, p<0.0001), but not for tones, or stress*tones (p>0.24). Post-hoc analysis using BH revealed a significantly lowered freezing response in LBN males compared to NS at T1-5 (F(1,18)=6.695, p=0.019), T6-10 (F(1,18)=8.84, p=0.012, and T11-15 (F(1,18)=13.12, p=0.006). **E)** No significant main effect was observed using the two-way ANOVA for the fear retrieval ITIs in females (p>0.73). **F)** No significant differences were observed between LBN and NS males between the different ITIs in the fear retrieval task; the two-way ANOVA did reveal a significant main effect for ITIs (F(2.54)=6.80, p=0.002), but not for stress (F(1,54)=0.20, p=0.66), or stress*ITIs (F(2,54)=0.28, p=0.76). Further post-hoc analysis using BH revealed no significant differences (p>0.57). The timelines are presented

as mean ± standard error of the mean and all individual samples as points. Panel A-F represent female NS (n=10, female LBN (n=10), male NS (10), male LBN (11).

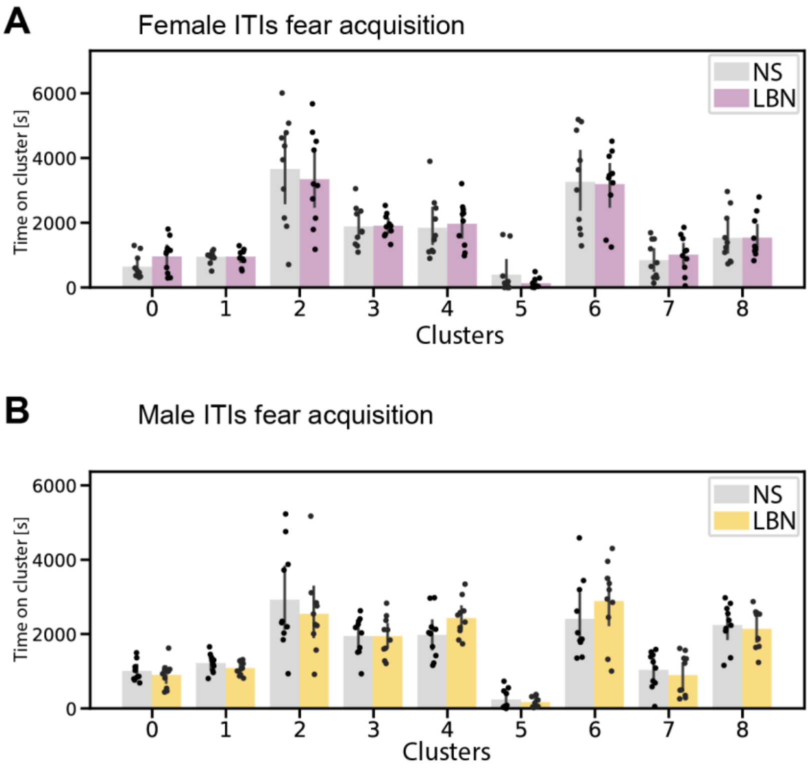

Supplemental Figure 3. Unsupervised clusters during the ITIs. A) Cluster enrichment for the female fear acquisition data using all four ITIs. No significant differences were observed using the independent samples t-test corrected for multiple testing using Benjamini-Hochberg's method across clusters (p>0.05). **B)** Cluster enrichment for the male fear acquisition data using all four ITIs. No significant differences were observed using the independent samples t-test corrected for multiple testing using Benjamini-Hochberg's method across clusters (p>0.05). Bar graphs represent mean ± standard deviation of the time proportion spent on each cluster. The timelines are presented as mean ± standard error of the mean and all individual samples as points. Panel A-F represent female NS (n=10, female LBN (n=10), male NS (10), male LBN (11).

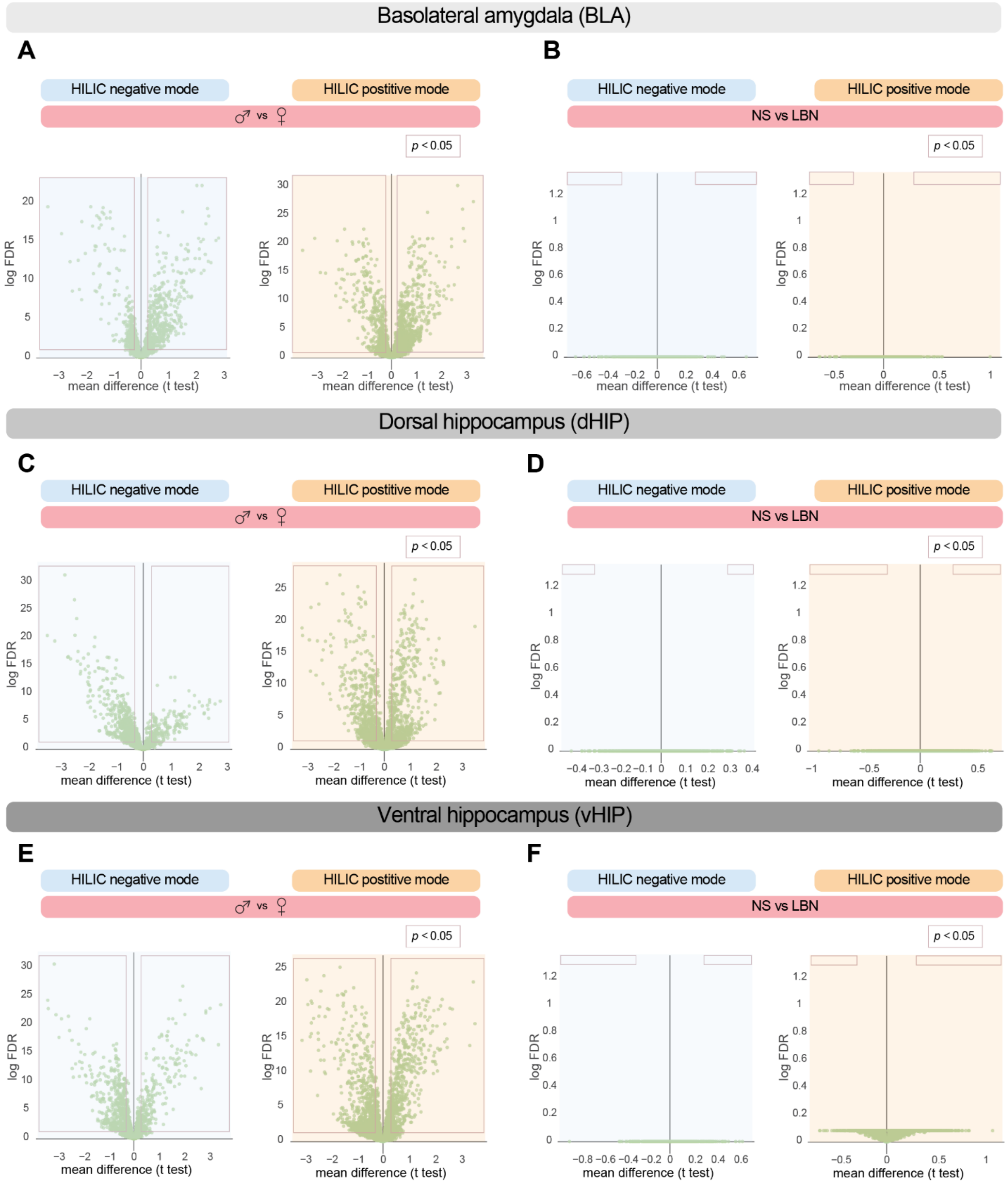

Supplemental Figure 4. Metabolomics differential expression analysis on sex and stress condition. Volcano plots show a large differential expression profile comparing male vs females regardless of stress condition in HILIC negative and positive mode, while no differential expression profile comparing NS and LBN conditions, regardless of sex in HILIC negative and positive mode in BLA **(A-B)**, dHIP **(C-D),** vHIP **(E-F)**. Panel A-F represent female NS (n=10, female LBN (n=10), male NS (n=10), male LBN (n=11).

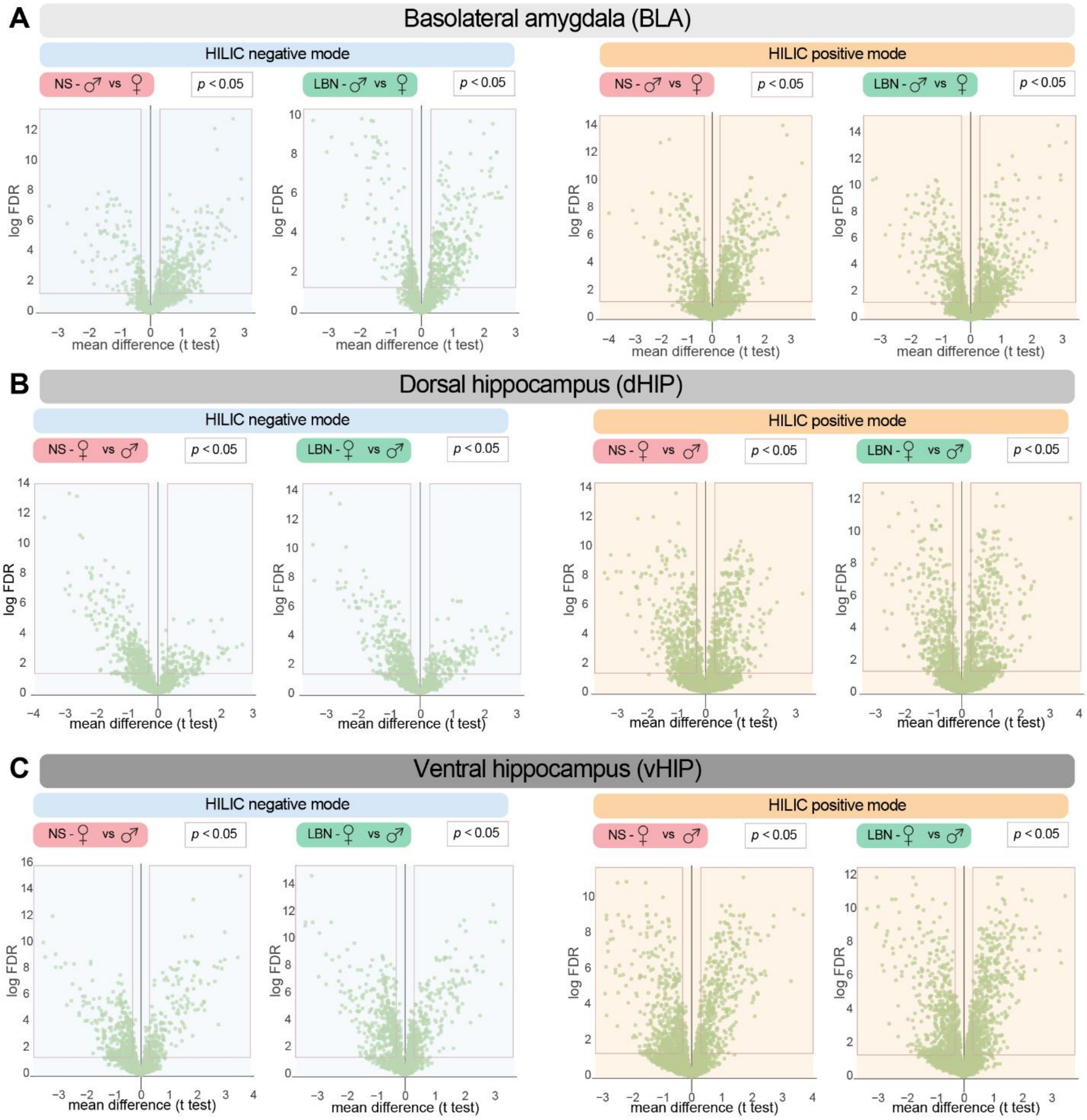

Supplemental Figure 5. Metabolomics differential expression analysis on the interaction between sex and stress condition. Volcano plots show a large differential expression profile comparing male vs females in NS, as well as LBN conditions in HILIC negative and positive mode in the BLA **(A)**, dHIP **(B)**, and vHIP **(C)**. Panel A-C represent female NS (n=10, female LBN (n=10), male NS (n=10), male LBN (n=11).
