# Supplementary material for: Sex-specific fear acquisition following early life stress is linked to amygdala and hippocampal purine and glutamate metabolism
Source: Commun Biol. 2024 Dec 20;7:1684. doi: 10.1038/s42003-024-07396-8 (PMC11659400; doi:10.1038/s42003-024-07396-8)
Supplement: Supplementary file 3 — Description of Additional Supplementary Materials [file 42003_2024_7396_MOESM3_ESM.pdf]

Description of Additional Supplementary Files

File name: Supplementary Data 1

Description: Numerical source data for all graphs in the manuscript

File name: Supplementary Data 2

Description: The lists of altered metabolites across the different brain regions

File name: Supplementary Video

Description: Example video fragments of the different unsupervised clusters, labeled in the video per cluster.
