# Supplementary material for: Sex-specific fear acquisition following early life stress is linked to amygdala and hippocampal purine and glutamate metabolism
Source: Commun Biol. 2024 Dec 20;7:1684. doi: 10.1038/s42003-024-07396-8 (PMC11659400; doi:10.1038/s42003-024-07396-8)
Supplement: Supplementary file 5 — Supplementary Data 2 [file 42003_2024_7396_MOESM5_ESM.pdf]

Supplemental data 2. HILIC metabolites lists for BLA, dHip and vHIP.

BLA - Male NS vs Female NS		
Metabolites HILIC Negative	Metabolites HILIC Negative	Metabolites HILIC Negative
Adenine	Adenosine monophosphate	gamma-butyrobetaine
Adenosine monophosphate	Leucine	GDP-deoxyhexose
NAD	Isoleucine	GDP-hexose
pentose	HMDB0240771	hexose phosphate
O-Phosphoethanolamine	Hypoxanthine	Adenine
Inosine	Citicoline	Adenosine monophosphate
Adenosine	NAD	Adenosine
Oxidized glutathione	Guanosine	Cytidine monophosphate
Xanthine	Adenosine triphosphate	Guanine
HMDB0245277	Adenosine	Guanosine
ADP	LPE(20:4)	Glutamic acid
Uridine	Uridine	Hypoxanthine
UDP-glucose	ADP	L-Alanine
pentose phosphate	Oxidized glutathione	L-Asparagine
N-gamma-Glutamylglutamine	LPE(18:1)	Isoleucine
Cytidine monophosphate	Butyrylcarnitine	Serine
PA(32:0)	Inosine	L-Aspartic acid
PG(36:1)	N-gamma-Glutamylglutamine	Inosine
Dodecanoic acid	Uridine diphosphate glucose	Palmitoylcarnitine
D-Sedoheptulose 7-phosphate	Niacinamide	O-Phosphoethanolamine
Succinic acid	L-Aspartic acid	Succinic acid
Pyroglutamic acid	Hexanoylcarnitine	Phosphoenolpyruvic acid
monohydroxybenzoic acid	Stearoylcarnitine	Pyroglutamic acid
Uridine 5'-monophosphate	Xanthine	Uridine diphosphate glucose
GDP-deoxyhexose	S-Adenosylhomocysteine	Uridine 5'-monophosphate
UDP-N-acetyl-hexosamine	Uridine 5'-diphosphate	Xanthine
N-Acetyl-L-glutamic acid	gamma-butyrobetaine	Uridine 5'-diphosphate
PE(36:1)	Creatinine	Uridine
GDP-hexose	LPC(16:0)	Adenosine triphosphate
LPE(18:1)	L-Alanine	Creatinine
hexose-phosphate	UDP-N-acetyl-hexosamine	Dodecanoic acid
L-Aspartic acid	L-Asparagine	Glutamine
	LPC(18:1)	Leucine
	Trimethylamine	Hexanoylcarnitine
	Guanosine monophosphate	Stearoylcarnitine
	Cytidine monophosphate	NAD
	Glutamic acid	Trimethylamine

	hexose phosphate	Uridine diphosphate glucuronic acid
	Uridine diphosphate glucuronic acid	S-Adenosylhomocysteine
	Phosphoenolpyruvic acid	D-Sedoheptulose 7-phosphate
	Serine	N-Acetyl-L-glutamic acid
	Guanine	ADP
	Palmitoylcarnitine	Guanosine monophosphate
	HMDB0304563	Niacinamide
	Glutamine	Citicoline
	PC(18:2)	Butyrylcarnitine
		Oxidized glutathione
		N-gamma-Glutamylglutamine
		HMDB0240771
		HMDB0245277
		HMDB0304563
		LPC(16:0)
		LPC(18:1)
		LPE(18:1)
		LPE(20:4)
		monohydroxybenzoic acid
		PA(32:0)
		PC(18:2)
		PE(36:1)
		pentose
		pentose phosphate
		PG(36:1)
		UDP-N-acetyl-hexosamine

BLA - Male LBN vs Female LBN		
**Metabolites HILIC Negative**	**Metabolites HILIC Positive**	**Merged metabolites**
Adenosine	Adenosine	Disaccharide
Adenine	NAD	gamma-butyrobetaine
Adenosine monophosphate	Citicoline	GDP-deoxyhexose
Inosine	Guanine	GDP-hexose
NAD	Butyrylcarnitine	hexose phosphate
Xanthine	Hypoxanthine	Carnosine
pentose	Guanosine	Adenine
O-Phosphoethanolamine	Inosine	Adenosine monophosphate
Oxidized glutathione	Oxidized glutathione	Adenosine
pentose phosphate	Adenosine triphosphate	Cytidine monophosphate
Cytidine monophosphate	N-gamma-Glutamylglutamine	Guanine
Uridine 5'-monophosphate	LPE(18:1)	Guanosine
PG(36:1)	Hexanoylcarnitine	Hypoxanthine
ADP	Uridine diphosphate glucose	L-Asparagine
Uridine	L-Asparagine	Isoleucine
Succinic acid	LPE(20:4)	L-Aspartic acid
Adenosine triphosphate	Uridine 5'-diphosphate	Inosine
UDP-N-acetyl-hexosamine	Xanthine	Pantothenic acid
D-Sedoheptulose 7-phosphate	UDP-N-acetyl-hexosamine	O-Phosphoethanolamine
monohydroxybenzoic acid	Stearoylcarnitine	Succinic acid
Pyroglutamic acid	Niacinamide	Pyroglutamic acid
N-gamma-Glutamylglutamine	Phosphorylcholine	Uridine diphosphate glucose
PA(32:0)	Guanosine diphosphate	Uridine 5'-monophosphate
GDP-deoxyhexose	ADP	Xanthine
GDP-hexose	gamma-butyrobetaine	Uridine 5'-diphosphate
CDP	Leucine	Uridine
Homocarnosine	Citrulline	Adenosine triphosphate
LPE(18:1)	Pantothenic acid	Creatinine
PE(38:4)	Creatinine	Glutamine
Carnosine	Uridine diphosphate glucuronic acid	Leucine
Methionine	Guanosine monophosphate	Isovalerylcarnitine
Uridine diphosphate glucuronic acid	hexose phosphate	Methionine
PE(36:1)	Cytidine monophosphate	Homocarnosine
	L-Aspartic acid	Hexanoylcarnitine
	Guanosine triphosphate	Stearoylcarnitine
	Uridine	NAD
	Isovalerylcarnitine	Citrulline
	Adenosine monophosphate	Uridine diphosphate glucuronic acid

	Uridine 5'-monophosphate	D-Sedoheptulose 7-phosphate
	Glutamine	Guanosine diphosphate
	Disaccharide	Guanosine triphosphate
		ADP
		Guanosine monophosphate
		Niacinamide
		Citicoline
		CDP
		Phosphorylcholine
		Butyrylcarnitine
		Oxidized glutathione
		N-gamma-Glutamylglutamine
		HMDB0245277
		LPE(18:1)
		LPE(20:4)
		monohydroxybenzoic acid
		PA(32:0)
		PE(36:1)
		PE(38:4)
		pentose
		pentose phosphate
		PG(36:1)
		UDP-N-acetyl-hexosamine

dHIP - Male NS vs Female NS		
**Metabolites HILIC Negative**	**Metabolites HILIC Negative**	**Metabolites HILIC Negative**
4-Decylbenzenesulfonic acid	Cysteineglutathione disulfide	4-Decylbenzenesulfonic acid
4-Dodecylbenzenesulfonic Acid	4-Trimethylammoniobutanoic acid	4-Dodecylbenzenesulfonic Acid
Adenine	Acetyl-D-carnitine	Adenine
Adenosine	Adenosine	Adenosine
Adenosine monophosphate	Adenosine monophosphate	Adenosine monophosphate
ADP	Adenosine triphosphate	ADP
AICAR	ADP	AICAR
Ascorbic acid	Anserine	Ascorbic acid
C(18:1)	Betaine	C(18:1)
Cytidine monophosphate	Butyrylcarnitine	Cytidine monophosphate
gamma-Glutamylglutamic acid	Carnosine	gamma-Glutamylglutamic acid
Glutamic acid	Cytidine monophosphate	Glutamic acid
Hypoxanthine	Glutamic acid	Hypoxanthine
Inosine	Glutamine	Inosine
Inosinic acid	Glutaminylglutamic acid	Inosinic acid
LPC(15:2)	Glutathione	LPC(15:2)
LPE(18:1)	Guanine	LPE(18:1)
LPE(20:4)	Guanosine	LPE(20:4)
monohydroxybenzoic acid	Guanosine diphosphate	monohydroxybenzoic acid
N-Undecylbenzenesulfonic acid	Guanosine monophosphate	N-Undecylbenzenesulfonic acid
NAD	Histidine	NAD
Oxidized glutathione	Inosine	Oxidized glutathione
PA(32:0)	L-Aspartic acid	PA(32:0)
Pantothenic acid	LPC(16:0)	Pantothenic acid
pentose	LPC(18:1)	pentose
pentose phosphate	LPC(18:2)	pentose phosphate
PG(36:1)	LPE(18:1)	PG(36:1)
PS(36:1)	LPE(20:4)	PS(36:1)
Xanthine	NAD	Xanthine
	Oleoylcarnitine	Cysteineglutathione disulfide
	Oxidized glutathione	4-Trimethylammoniobutanoic acid
	Palmitoylcarnitine	Acetyl-D-carnitine
	Tributyl Phosphate	Adenosine triphosphate
	Uridine	Anserine
	Uridine 5'-diphosphate	Betaine
	Uridine diphosphate glucose	Butyrylcarnitine

	Uridine diphosphate-N-acetylgalactosamine	Carnosine
	Urocanic acid	Glutamine
	Xanthine	Glutaminylglutamic acid
		Glutathione
		Guanine
		Guanosine
		Guanosine diphosphate
		Guanosine monophosphate
		Histidine
		L-Aspartic acid
		LPC(16:0)
		LPC(18:1)
		LPC(18:2)
		Oleoylcarnitine
		Palmitoylcarnitine
		Tributyl Phosphate
		Uridine
		Uridine 5'-diphosphate
		Uridine diphosphate glucose
		Uridine diphosphate-N-acetylgalactosamine
		Urocanic acid

dHIP - Male LBN vs Female LBN		
Metabolites HILIC Negative	Metabolites HILIC Negative	Metabolites HILIC Negative
4-Decylbenzenesulfonic acid	4-Trimethylammoniobutanoic acid	4-Decylbenzenesulfonic acid
4-Dodecylbenzenesulfonic Acid	5'-Methylthioadenosine	4-Dodecylbenzenesulfonic Acid
Adenine	Acetyl-D-carnitine	4-Trimethylammoniobutanoic acid
Adenosine	Adenosine	5'-Methylthioadenosine
Adenosine monophosphate	Adenosine monophosphate	Acetyl-D-carnitine
ADP	Adenosine triphosphate	Adenine
AICAR	ADP	Adenosine
Ascorbic acid	Anserine	Adenosine monophosphate
Cytidine monophosphate	Butyrylcarnitine	Adenosine triphosphate
gamma-Glutamylglutamic acid	Carnosine	ADP
Glutamic acid	Cysteineglutathione disulfide	AICAR
hexose bisphosphate	Cytidine monophosphate	Anserine
Hypoxanthine	Cytosine	Ascorbic acid
Inosine	gamma-Glutamylglutamic acid	Butyrylcarnitine
Inosinic acid	Guanine	Carnosine
LPC(15:2)	Guanosine diphosphate	Cysteineglutathione disulfide
LPE(20:4)	Guanosine monophosphate	Cytidine monophosphate
monohydroxybenzoic acid	hexose phosphate	Cytosine
N-gamma-Glutamylglutamine	Histidine	gamma-Glutamylglutamic acid
N-Undecylbenzenesulfonic acid	Hypoxanthine	Glutamic acid
NAD	Inosine	Guanine
Oxidized glutathione	L-Aspartic acid	Guanosine diphosphate
PA(32:0)	LPC(16:0)	Guanosine monophosphate
pentose	LPC(18:1)	hexose bisphosphate
pentose phosphate	LPC(18:2)	hexose phosphate
PG(36:1)	LPE(18:1)	Histidine
PS(36:1)	LPE(20:4)	Hypoxanthine
Xanthine	LPE(38:5)	Inosine
	Myristoylcarnitine	Inosinic acid
	NAD	L-Aspartic acid
	Niacinamide	LPC(15:2)
	Oxidized glutathione	LPC(16:0)
	Uridine	LPC(18:1)
	Uridine diphosphate glucose	LPC(18:2)

	Uridine diphosphate-N-acetylgalactosamine	LPE(18:1)
	Urocanic acid	LPE(20:4)
	Xanthine	LPE(38:5)
		monohydroxybenzoic acid
		Myristoylcarnitine
		N-gamma-Glutamylglutamine
		N-Undecylbenzenesulfonic acid
		NAD
		Niacinamide
		Oxidized glutathione
		PA(32:0)
		pentose
		pentose phosphate
		PG(36:1)
		PS(36:1)
		Uridine
		Uridine diphosphate glucose
		Uridine diphosphate-N-acetylgalactosamine
		Urocanic acid
		Xanthine

vHIP - Male NS vs Female NS		
**Metabolites HILIC Negative**	**Metabolites HILIC Negative**	**Metabolites HILIC Negative**
4-Dodecylbenzenesulfonic Acid	5-(2-Hydroxyethyl)-4-methylthiazole	4-Dodecylbenzenesulfonic Acid
Adenine	5'-Methylthioadenosine	5-(2-Hydroxyethyl)-4-methylthiazole
Adenosine	Adenine	5'-Methylthioadenosine
Adenosine monophosphate	Adenosine	Adenine
Adenosine triphosphate	Adenosine monophosphate	Adenosine
ADP	Adenosine triphosphate	Adenosine monophosphate
Cytidine monophosphate	ADP	Adenosine triphosphate
Dodecanoic acid	Caprylic acid	ADP
gamma-Glutamylglutamic acid	Citicoline	Caprylic acid
Guanosine diphosphate	Cytidine monophosphate	Citicoline
Guanosine monophosphate	DL-Proline	Cytidine monophosphate
Hypoxanthine	GDP-hexose	DL-Proline
Inosine	Glutamic acid	Dodecanoic acid
LPC(15:2)	Glutamine	gamma-Glutamylglutamic acid
LPE(18:1)	Guanine	GDP-hexose
LPE(20:4)	Guanosine	Glutamic acid
O-Phosphoethanolamine	Guanosine diphosphate	Glutamine
PA(32:0)	Guanosine monophosphate	Guanine
PG(36:1)	Histamine	Guanosine
Succinic acid	Hypoxanthine	Guanosine diphosphate
Uridine	Inosine	Guanosine monophosphate
Uridine diphosphate glucose	Inosinic acid	Histamine
Uridine diphosphate glucuronic acid	LPC(18:1)	Hypoxanthine
Xanthine	LPE(18:1)	Inosine
	LPE(20:4)	Inosinic acid
	Methionine sulfoxide	LPC(15:2)
	N-Acetyl-L-aspartic acid	LPC(18:1)
	N-gamma-Glutamylglutamine	LPE(18:1)
	NAD	LPE(20:4)
	Oxidized glutathione	Methionine sulfoxide
	Pantothenic acid	N-Acetyl-L-aspartic acid
	Phosphorylcholine	N-gamma-Glutamylglutamine
	Pyroglutamic acid	NAD
	Tributyl Phosphate	O-Phosphoethanolamine
	UDP-N-acetyl-hexosamine	Oxidized glutathione
	Uridine	PA(32:0)

	Uridine 5'-monophosphate	Pantothenic acid
	Uridine diphosphate glucose	PG(36:1)
	Xanthine	Phosphorylcholine
		Pyroglutamic acid
		Succinic acid
		Tributyl Phosphate
		UDP-N-acetyl-hexosamine
		Uridine
		Uridine 5'-monophosphate
		Uridine diphosphate glucose
		Uridine diphosphate glucuronic acid
		Xanthine

vHIP - Male LBN vs Female LBN		
Metabolites HILIC Negative	Metabolites HILIC Negative	Metabolites HILIC Negative
4-Dodecylbenzenesulfonic Acid	5-(2-Hydroxyethyl)-4-methylthiazole	4-Dodecylbenzenesulfonic Acid
Adenine	Adenine	5-(2-Hydroxyethyl)-4-methylthiazole
Adenosine	Adenosine	Adenine
Adenosine monophosphate	Adenosine monophosphate	Adenosine
Adenosine triphosphate	Adenosine triphosphate	Adenosine monophosphate
ADP	ADP	Adenosine triphosphate
Cytidine monophosphate	Betaine	ADP
gamma-Glutamylglutamic acid	Caprylic acid	Betaine
Glutamic acid	Carnosine	Caprylic acid
guanosine 5'-diphospho-methylpentose	Citicoline	Carnosine
Guanosine diphosphate	Citrulline	Citicoline
Guanosine monophosphate	Cytidine monophosphate	Citrulline
hexose bisphosphate	gamma-Glutamylglutamic acid	Cytidine monophosphate
hexose phosphate	GDP-hexose	gamma-Glutamylglutamic acid
Hypoxanthine	Glutamic acid	GDP-hexose
Inosine	Glutamine	Glutamic acid
LPC(15:2)	Guanine	Glutamine
LPE(18:1)	Guanosine	Guanine
LPE(20:4)	Guanosine diphosphate	Guanosine
monohydroxybenzoic acid	Guanosine monophosphate	guanosine 5'-diphospho-methylpentose
O-Phosphoethanolamine	Hexanoylcarnitine	Guanosine diphosphate
Oxidized glutathione	Histidine	Guanosine monophosphate
PA(32:0)	Homocarnosine	Hexanoylcarnitine
pentose phosphate	Hypoxanthine	hexose bisphosphate
PG(36:1)	Inosine	hexose phosphate
Succinic acid	Inosinic acid	Histidine
Uridine	L-Aspartic acid	Homocarnosine
Uridine 5'-diphosphohexonic acid	LPC(16:0)	Hypoxanthine
Uridine 5'-monophosphate	LPC(18:1)	Inosine
Uridine diphosphate glucose	LPC(18:2)	Inosinic acid
Uridine diphosphate glucuronic acid	LPE(18:1)	L-Aspartic acid
Uridine diphosphate-N-acetylhexoseamine	LPE(20:4)	LPC(15:2)
Xanthine	Methionine sulfoxide	LPC(16:0)

	NAD	LPC(18:1)
	Oxidized glutathione	LPC(18:2)
	Phosphorylcholine	LPE(18:1)
	Phosphoserine	LPE(20:4)
	Pyroglutamic acid	Methionine sulfoxide
	Tributyl Phosphate	monohydroxybenzoic acid
	UDP-N-acetyl-hexoseamine	NAD
	Uridine	O-Phosphoethanolamine
	Uridine diphosphate glucose	Oxidized glutathione
		PA(32:0)
		pentose phosphate
		PG(36:1)
		Phosphorylcholine
		Phosphoserine
		Pyroglutamic acid
		Succinic acid
		Tributyl Phosphate
		UDP-N-acetyl-hexoseamine
		Uridine
		Uridine 5'-diphosphohexonic acid
		Uridine 5'-monophosphate
		Uridine diphosphate glucose
		Uridine diphosphate glucuronic acid
		Uridine diphosphate-N-acetylhexoseamine
		Xanthine
