# Supplementary material for: Sex-specific fear acquisition following early life stress is linked to amygdala and hippocampal purine and glutamate metabolism
Source: Commun Biol. 2024 Dec 20;7:1684. doi: 10.1038/s42003-024-07396-8 (PMC11659400; doi:10.1038/s42003-024-07396-8)
Supplement: Supplementary file 7 — Reporting summary [file 42003_2024_7396_MOESM7_ESM.pdf]

nature portfolio

Corresponding author(s): Mathias V. Schmidt

Last updated by author(s): Nov 29, 2024

Reporting Summary

Nature Portfolio wishes to improve the reproducibility of the work that we publish. This form provides structure for consistency and transparency in reporting. For further information on Nature Portfolio policies, see our Editorial Policies and the Editorial Policy Checklist.

Statistics

For all statistical analyses, confirm that the following items are present in the figure legend, table legend, main text, or Methods section.

n/a	Confirmed	
☐	☒	The exact sample size (*n*) for each experimental group/condition, given as a discrete number and unit of measurement
☐	☒	A statement on whether measurements were taken from distinct samples or whether the same sample was measured repeatedly
☐	☒	The statistical test(s) used AND whether they are one- or two-sided<br>*Only common tests should be described solely by name; describe more complex techniques in the Methods section.*
☒	☐	A description of all covariates tested
☐	☒	A description of any assumptions or corrections, such as tests of normality and adjustment for multiple comparisons
☐	☒	A full description of the statistical parameters including central tendency (e.g. means) or other basic estimates (e.g. regression coefficient) AND variation (e.g. standard deviation) or associated estimates of uncertainty (e.g. confidence intervals)
☐	☒	For null hypothesis testing, the test statistic (e.g. *F*, *t*, *r*) with confidence intervals, effect sizes, degrees of freedom and *P* value noted<br>*Give P values as exact values whenever suitable.*
☒	☐	For Bayesian analysis, information on the choice of priors and Markov chain Monte Carlo settings
☒	☐	For hierarchical and complex designs, identification of the appropriate level for tests and full reporting of outcomes
☒	☐	Estimates of effect sizes (e.g. Cohen's *d*, Pearson's *r*), indicating how they were calculated

Our web collection on statistics for biologists contains articles on many of the points above.

Software and code

Policy information about availability of computer code

Data collection	All filmed videos were processed independently with the automated video-tracking software AnyMaze 6.33 (fear conditioning freezing analysis) and DeepLabCut 2.2b7 (for pose estimation prior to DeepOF processing).
Data analysis	After pose estimation, data analysis was carried out with our custom DeepOF module v0.4.6. These tasks included motion tracking preprocessing, and unsupervised analyses of behavior. Reported statistical analyses and graphs were done in RStudio (with R 4.1.1) and python (v 3.9.13).

For manuscripts utilizing custom algorithms or software that are central to the research but not yet described in published literature, software must be made available to editors and reviewers. We strongly encourage code deposition in a community repository (e.g. GitHub). See the Nature Portfolio guidelines for submitting code & software for further information.

Data

Policy information about availability of data

All manuscripts must include a data availability statement. This statement should provide the following information, where applicable:

- Accession codes, unique identifiers, or web links for publicly available datasets
- A description of any restrictions on data availability
- For clinical datasets or third party data, please ensure that the statement adheres to our policy

All data and the accompanying code to perform the analyses and creating the figures are available via a open-source repositories, specified in the data availability statement.

Research involving human participants, their data, or biological material

Policy information about studies with human participants or human data. See also policy information about sex, gender (identity/presentation), and sexual orientation and race, ethnicity and racism.

Reporting on sex and gender	n.a.
Reporting on race, ethnicity, or other socially relevant groupings	n.a.
Population characteristics	n.a.
Recruitment	n.a.
Ethics oversight	n.a.

Note that full information on the approval of the study protocol must also be provided in the manuscript.

Field-specific reporting

Please select the one below that is the best fit for your research. If you are not sure, read the appropriate sections before making your selection.

☒ Life sciences ☐ Behavioural & social sciences ☐ Ecological, evolutionary & environmental sciences

For a reference copy of the document with all sections, see nature.com/documents/nr-reporting-summary-flat.pdf

Life sciences study design

All studies must disclose on these points even when the disclosure is negative.

Sample size	Given the exploratory nature of the analyses, no sample size was calculated a priori. A sample size of at least 10 animals per condition was established for the means per group to be approximately normal according to the central limit theorem.
Data exclusions	No data were excluded from data analysis
Replication	Unsupervised models obtained with DeepOF were internally validated using cross validation in the same dataset. Independent datasets were not included at this stage in the manuscript.
Randomization	The mice were randomly divided at postnatal day 2 into the ELS condition or the non-stressed condition.
Blinding	During data collection, the investigators were not blinded to group allocation. However, investigators were passively involved in data collection, as animals were left undisturbed in the experimental environment during testing. During data analysis, investigators were blinded for group allocation.

Reporting for specific materials, systems and methods

We require information from authors about some types of materials, experimental systems and methods used in many studies. Here, indicate whether each material, system or method listed is relevant to your study. If you are not sure if a list item applies to your research, read the appropriate section before selecting a response.

Materials & experimental systems

n/a	Involved in the study
☒	☐ Antibodies
☒	☐ Eukaryotic cell lines
☒	☐ Palaeontology and archaeology
☐	☒ Animals and other organisms
☒	☐ Clinical data
☒	☐ Dual use research of concern
☒	☐ Plants

Methods

n/a	Involved in the study
☒	☐ ChIP-seq
☒	☐ Flow cytometry
☒	☐ MRI-based neuroimaging

Animals and other research organisms

Policy information about studies involving animals; ARRIVE guidelines recommended for reporting animal research, and Sex and Gender in Research

Laboratory animals	C57Bl/6N male and female mice at 2 months of age
Wild animals	n.a.
Reporting on sex	All findings were tested in female and male animals and specific sex effects are reported
Field-collected samples	n.a.
Ethics oversight	All experiments are in accordance with the European Communities Council Directive 2010/63/EU

Note that full information on the approval of the study protocol must also be provided in the manuscript.

Plants

Seed stocks	n.a.
Novel plant genotypes	n.a.
Authentication	n.a.
